# Targeting Triple Negative Breast Cancer With Oncolytic Adenoviruses

**DOI:** 10.3389/fmolb.2022.901392

**Published:** 2022-06-24

**Authors:** Gabriela Green-Tripp, Callum Nattress, Gunnel Halldén

**Affiliations:** ^1^ Centre for Biomarkers and Biotherapeutics, Barts Cancer Institute, Queen Mary University of London, London, United Kingdom; ^2^ Cell Communication Lab, Department of Oncology, University College London Cancer Institute, London, United Kingdom

**Keywords:** tumour-selective, novel therapies, lysis, immune activation, metastatic breast cancer, OAd

## Abstract

Breast cancer (BC) is the most common cancer globally, accounting for 685,000 deaths in 2020. Triple-negative breast cancers (TNBC) lack oestrogen (ER) and progesterone (PR) hormone receptor expression and HER2 overexpression. TNBC represent 10–15% of all BC with high incidence in women under 50-years old that have *BRCA* mutations, and have a dismal prognosis. African American and Hispanic women are at higher risk partly due to the common occurrence of *BRCA* mutations. The standard treatment for TNBC includes surgery, radiotherapy, and chemotherapy although, resistance to all standard-of-care therapies eventually develops. It is crucial to identify and develop more efficacious therapeutics with different mechanisms of action to improve on survival in these women. Recent findings with oncolytic adenoviruses (OAds) may generate a new strategy to improve on the outcomes for women afflicted by TNBC and other types of BC. OAds are genetically engineered to selectively lyse, eliminate and recruit the host antitumour immune responses, leaving normal cells unharmed. The most common modifications are deletions in the early gene products including the E1B55 KDa protein, specific regions of the E1A protein, or insertion of tumour-specific promoters. Clinical trials using OAds for various adenocarcinomas have not yet been sufficiently evaluated in BC patients. Preclinical studies demonstrated efficacy in BC cell lines, including TNBC cells, with promising novel adenoviral mutants. Here we review the results reported for the most promising OAds in preclinical studies and clinical trials administered alone and in combination with current standard of care or with novel therapeutics. Combinations of OAds with small molecule drugs targeting the epidermal growth factor receptor (EGFR), androgen receptor (AR), and DNA damage repair by the novel PARP inhibitors are currently under investigation with reported enhanced efficacy. The combination of the PARP-inhibitor Olaparib with OAds showed an impressive anti-tumour effect. The most promising findings to date are with OAds in combination with antibodies towards the immune checkpoints or expression of cytokines from the viral backbone. Although safety and efficacy have been demonstrated in numerous clinical trials and preclinical studies with cancer-selective OAds, further developments are needed to eliminate metastatic lesions, increase immune activation and intratumoural viral spread. We discuss shortcomings of the OAds and potential solutions for improving on patient outcomes.

## Introduction

**FIGURE 1:**
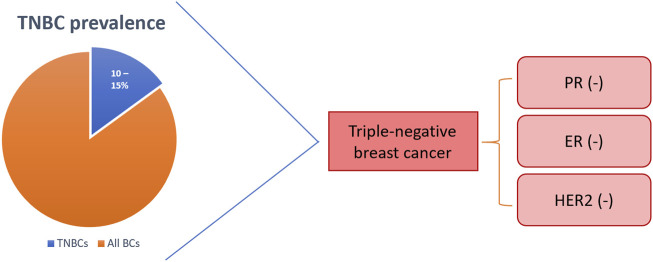
Triple-negative breast cancer (TNBC) is hormone receptor-negative with poor or no HER2-expression. TNBC represents 10–15% of all breast cancers. BCs: breast cancers; PR: progesterone receptor; ER: oestrogen receptor; HER2: human epidermal growth factor receptor 2.

Breast cancer (BC) is the most prevalent female cancer worldwide with over 2.1 million women diagnosed and over 620,000 BC-related deaths in 2018 (Sharma, 2021). BC is commonly divided into three groups, 1) luminal BC that express the oestrogen (ER) and progesterone (PR) receptors, 2) basal BC that overexpress human epidermal growth factor receptor 2 (HER2), and 3) basal triple-negative BC (TNBC) that does not express any of the three receptors (Figure 1) (Goldhirsch et al., 2011; Uscanga-Perales et al., 2019). The TNBC subtype is the most aggressive and has the poorest prognosis of all BC subtypes although, it is the least common, constituting only 10–15% of cases (Pal et al., 2014). In fact, 5% of all-cancer-related deaths are characterised as TNBC every year (Adel, 2021). Current therapeutics for BC have limited efficacy in TNBC patients since hormonal therapies have no effect and resistance to cytotoxic drugs rapidly develops.

Younger premenopausal women that have *BRCA* mutations and those of African or Hispanic descent are at the highest risk of developing TNBC (Yao et al., 2017). African-American women are twice as likely and Hispanic women are 1.3 times more likely to develop TNBC than white and non-Hispanic women, respectively (Howlader et al., 2014). The increased frequency in these ethnic groups is associated with obesity and, in the Mexican-American population particularly, there is a high prevalence of *BRCA* mutations (25%) (Kwan et al., 2009; Gaudet et al., 2011; Weitzel et al., 2013). It has been demonstrated that all women with *BRCA1* mutations have a higher risk of developing TNBC (57%) (Atchley et al., 2008). In addition to *BRCA* mutations, dysregulation of additional signalling pathways and transcription factors have been indicated to play a role in the development of TNBC (Table 1).

**TABLE 1:** Dysregulated pathways and factors associated with TNBC.

Altered pathway/factor	Function	References
EGFR	Overexpression contributes to deregulated cell proliferation	Williams et al. (2015)
MAPK signalling pathway	Overactivation promotes uncontrolled cell proliferation and resistance to cell death	Qi et al. (2019)
PI3K/AKT/mTOR signalling pathway	Overexpression of mTOR (40–70%) and PIK3CA mutations (∼22%) deregulates cancer cell proliferation	(Costa et al., 2018; Qi et al., 2019)
*BRCA1* gene	Mutations render the protein defective in DNA damage repair in the majority of TNBC	Atchley et al. (2008)
Cancer-associated transcription factors (TFs)	Dysregulation of TMPRSS2, ETS, KLF4 and KLF5 promotes uncontrolled cell proliferation	Qi et al. (2019)

EGFR, epidermal growth factor receptor; MAPK, mitogen-activated protein kinase; PI3K, phosphatidylinositol 3-kinas;, PIK3CA, phosphatidylinositol-4, 5-bisphosphate 3-kinase catalytic subunit alpha; AKT, protein kinase B; mTOR, mammalian target of rapamycin; BRCA1, Breast cancer gene 1; TMPRSS2, transmembrane serine protease 2; ETS, E26 transformation-specific; KLF4, Krüppel-like factor 4; KLF5, Krüppel-like factor 5.

## Current Treatments for TNBC

**FIGURE 2:**
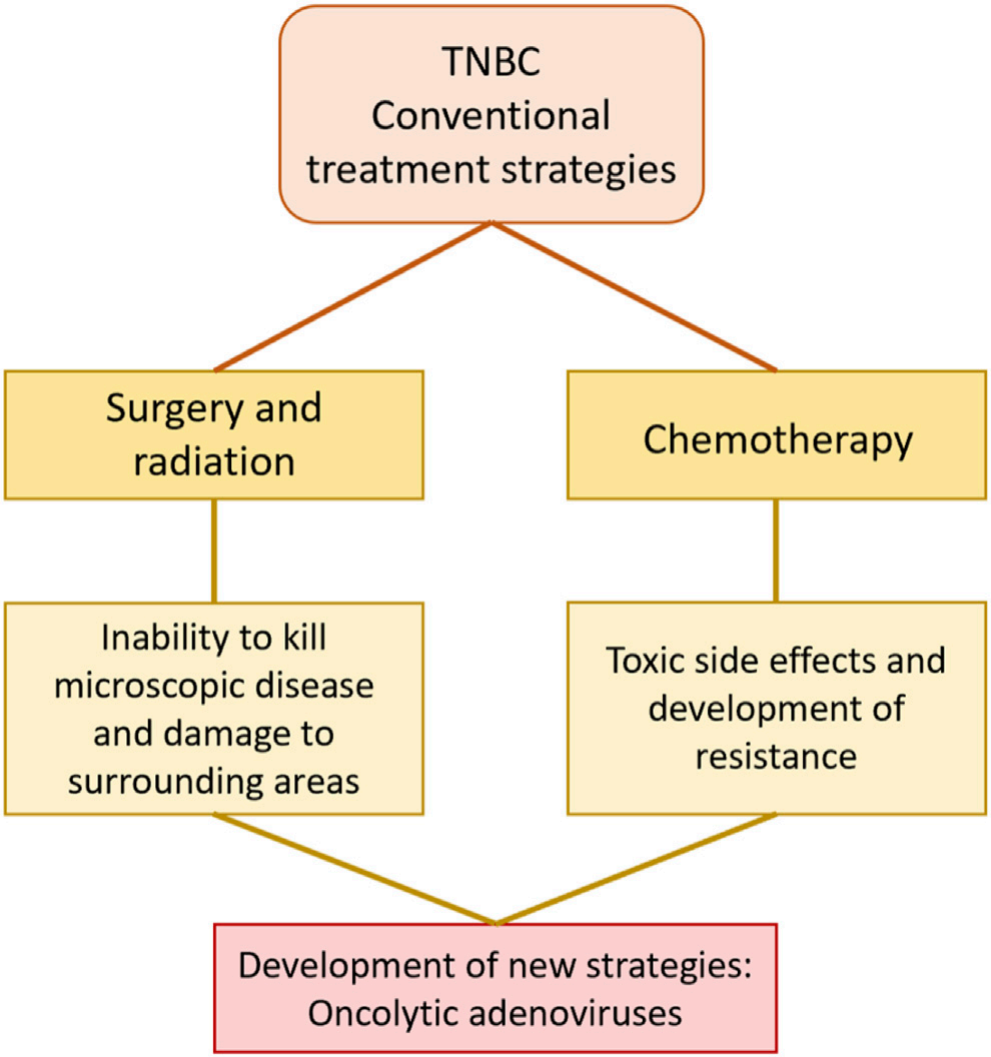
Conventional treatment strategies for TNBC include surgery, radiation, and chemotherapy but have limited efficacy. The poor outcomes of current therapeutics highlight the need for the development of novel strategies. Oncolytic adenoviruses are one of the most promising novel therapeutics for solid cancers including TNBC.

The current therapeutic approaches to treat TNBC are surgery, radiotherapy, chemotherapy, and a combination of these (Wahba and El-Hadaad, 2015). Surgery and radiotherapy are typical treatments in early stages (I-III) of the disease while chemotherapy is the choice at late-stage (IV) disease, when the cancer has already metastasised. However, aside from surgical resection of the primary tumour, current therapeutics are rarely curative and poor outcomes with rapid progression of the cancer result in high morbidity, mortality and early deaths. Therefore, novel therapeutic strategies are urgently needed (Figure 2). One promising strategy is the development of oncolytic adenoviruses (OAds). OAds have proven safety and efficacy in several clinical trials targeting solid cancers including BC and cancers with similar pathway alterations as seen in TNBC patients (Cody and Hurst, 2015; Nattress and Halldén, 2018; Bazan-Peregrino et al., 2021).

## Oncolytic Adenoviruses

**FIGURE 3:**
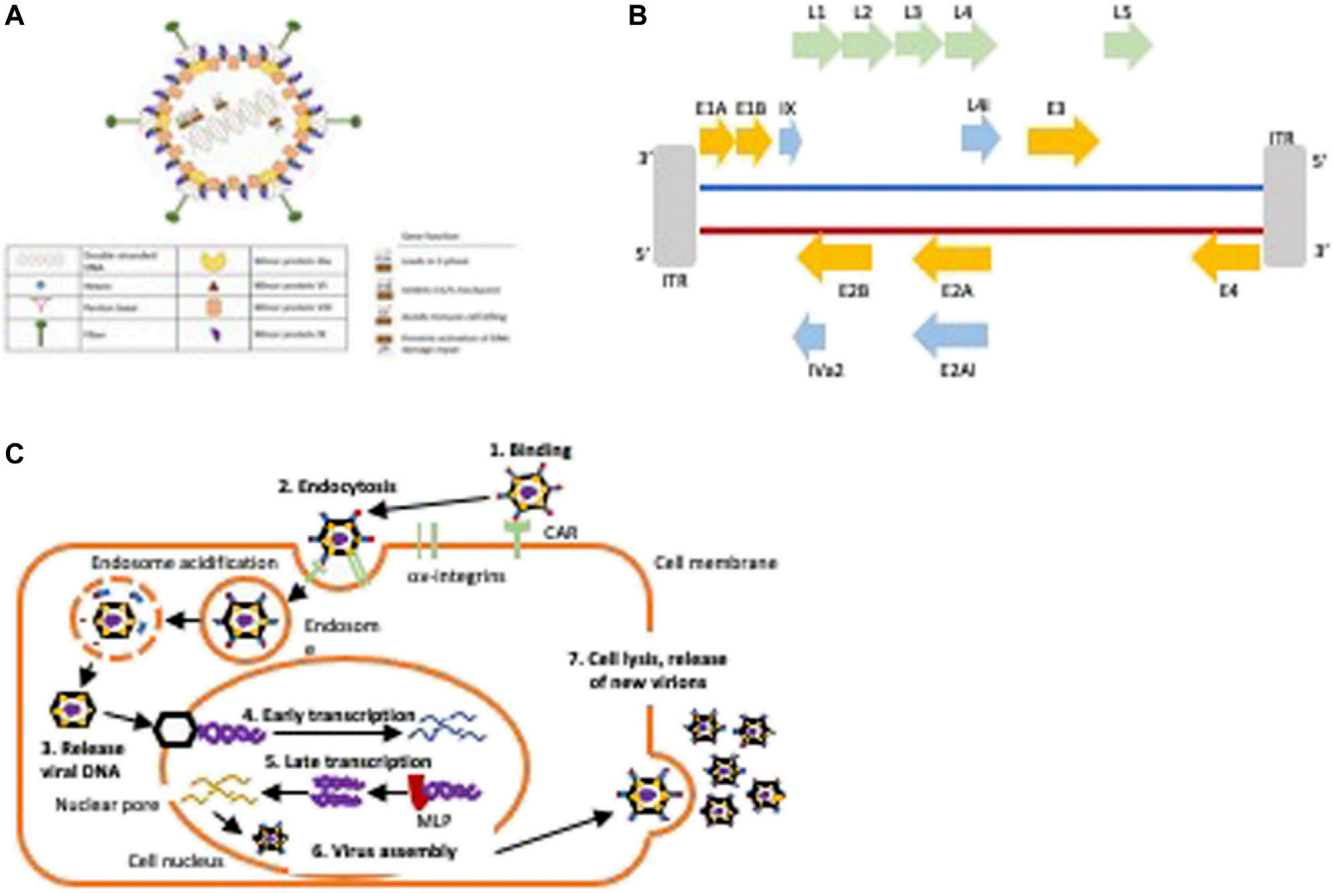
Structure and life cycle of HAdV5. **(A)** Hexon is the major coat protein, the fiber protein binds to the Coxsackie and adenovirus receptor (CAR), the penton base is essential for viral uptake through the endosome by binding to cellular αvβ3-and αvβ5-integrins, the minor proteins are essential for capsid stabilisation and in viral genome stability. Gene functions for early expressed viral genes (E1-E4) are indicated. **(B)** Schematic of the viral genome. The first protein to be expressed is E1A that leads to S-phase induction by binding pRb and releasing E2F. E1A initiates the transcription of other early genes including the E1B55K that inhibit premature apoptosis by binding to p53, the E1B19K that prevents death receptor mediated apoptosis, the E3 genes that prevent immune-mediated cell killing of infected cells, and the E4 genes that prevent activation of DNA damage repair responses. The E2A and E2B proteins are essential for viral DNA synthesis and amplification. The late genes (L1-5) are transcribed after activation of the major late promoter (MLP) to enable assembly of new virions. Strategies to engineer OAds include deletion of early viral genes or specific gene regions that are essential for viral propagation in normal cells, for example, the pRb-binding E1ACR2-domain or the p53-binding E1B55K gene or insertion of tumour-specific promoters to drive early E1 gene expression. E3 gene regions are frequently deleted to enable insertion of transgenes. **(C)** Illustration of the HAdV5 life cycle.

Human adenoviruses (HAdV) are small DNA viruses with a linear double stranded DNA (dsDNA; 35–40 kb) encapsulated by a protein coat (Figures 3A,B). The HAdV family is well characterised with seven serotypes and over 100 genotypes (Baker et al., 2018). In the majority of clinical trials, viruses of serotype C subtype 5 (HAdV5) have been employed due to the ease of genetic engineering of the viral genome, feasibility of large scale GMP production and proven safety in cancer patients, as well as their innate tropism for adenocarcinomas. The HAdV5 genome and its functions are well understood and can easily be engineered to replicate and kill cancer cells selectively with no toxicity to surrounding healthy tissue. Numerous OAd mutants have been engineered and evaluated in early phase clinical trials and were reported to have promising efficacy with only self-limiting side-effects. The main strategies by which HAdV5 are engineered to selectively replicate in tumour cells are: 1) deletion of genes that are necessary for viral replication in normal cells and are expendable in cancer cells with already deregulated cell cycle and signalling pathways, 2) insertion of tumour specific promoters such as hormone response elements, and 3) a combination of these approaches and/or insertion of cytotoxic transgenes to enhance anti-cancer efficacy (McConnell and Imperiale, 2004; Baker et al., 2018; Nattress and Halldén, 2018) (Table 2).

**TABLE 2:** Common deletions in engineered Oncolytic Adenoviruses (OAds).

Mutation and function	Virus	References
Deletion of E1B55 KDa protein to prevent p53-binding in normal cells	Onyx-015	(Nemunaitis et al., 2007) (Heise et al., 1997)
Deletion of E1ACR2 domain to prevent pRb-binding and S-phase entry in normal cells	∆24	Fueyo et al. (2000)
	*dl*922-947	Heise et al. (2000)
	DNX-2401	Lang et al. (2018)
	ORCA-010	Dong et al. (2014)
Replacing the E1A constitutive promoter with tumour specific promoters	OBP-301 (Telomelysin)	Nemunaitis et al. (2010)
Deletion of E3gp19K immunomodulatory genes to allow MHC class I antigen presentation	ORCA-010	Dong et al. (2014)

The first step in HAdV-infection is binding of the fibre knob to the Coxsackie and Adenovirus Receptor (CAR) on the epithelial cell surface (Figure 3C) (Baker et al., 2018). This leads to binding of the viral penton protein to αvß3-and αvß5-integrins followed by clathrin-dependent endocytosis and transport of the viral DNA to the nucleus along microtubules (Figure 3C). The first gene to be expressed is E1A that is an absolute requirement for expression of other viral genes and for viral replication. E1A forces the cell into S-phase by E1ACR2-binding to pRb leading to the release of E2F followed by S-phase entry and expression of additional early viral genes (Öberg et al., 2010; Baker et al., 2018). Subsequently, the E1B55K and E1B19K genes are expressed to inhibit the G1/S checkpoint activation in order to protect the infected cell from premature apoptosis (Leitner et al., 2009; Öberg et al., 2010). The E3 genes are expressed to avoid immune-mediated cell killing and E4 proteins prevent activation of the DNA damage repair (Wang et al., 2003; Cherubini et al., 2011).

There are a variety of underlying mechanisms, both direct and indirect, in which OAds can lead to anti-tumour efficacy in patients. Following initial infection, OAds naturally lead to oncolysis of tumour cells following sustained cancer-selective replication; this oncolytic process is vital in the replication cycle of OAds as it allows the release and spread of progeny virions to neighbouring cells throughout the tumour microenvironment (TME) (Mathis et al., 2005). Aside from direct oncolysis, OAds can also kill cancer cells *via* immunogenic cell death, which is an attractive property of OAd cancer therapy when considering the naturally immunosuppressive TME (Dunn et al., 2002). Aside from the release of virions, lysed cancer cells also release immunogenic factors including tumour-associated antigens (TAAs) and pathogen- and damage-associated molecular patterns to resident antigen presenting cells (APCs) of the TME. Following migration of APCs to draining lymph nodes and the successful cross-presentation of antigens, activation of anti-tumour immune responses secondary to OAd oncolysis can ensue, particularly by CD8^+^ T cells (Nattress and Halldén, 2018). It has recently been shown that OAds can decrease tumour-infiltrating CD8^+^ T cell exhaustion in patients with metastatic cancer following systemic OAd treatment. This reduction in exhaustion was also associated with improved overall survival of the patients (Liikanen et al., 2022). In one preclinical model, OAds were also shown to induce tumour-specific memory CD8^+^ T cell responses, meaning that patients receiving OAd therapy may benefit from anti-tumour immune responses to future recurrent disease (Chen et al., 2021). The most studied and clinically promising OAds to date are listed in Table 2 and described below.

## Clinical Trials With Oncolytic Adenoviruses Including BC Patients

### Deletion of the p53-Binding E1B55 KDa Protein in Onyx-015

The first OAd to enter clinical trials was Onyx-015 which contains deletions in the E1B55K gene and in the E3B domain (Khuri et al., 2000; Kirn, 2001). Expression of E1B55K, driven by E1A, inhibits cellular p53 to prevent premature apoptosis and to enable S-phase entry and viral propagation (Nemunaitis et al., 2000). Mutants deleted in E1B55K cannot complete a productive lifecycle in normal healthy cells while in cancer cells with dysregulated p53 pathway viral genome amplification, gene expression and assembly of virions will proceed. The immunomodulatory E3B genes were also deleted in Onyx-015 as an added safety feature in these early clinical trials (Heise et al., 1997; Nemunaitis et al., 2000). The Onyx-015 mutant was evaluated in numerous early phase clinical trials targeting solid tumours and was reported to be safe but had limited efficacy as a single agent while efficacy was improved in combination with chemotherapeutic drugs (Larson et al., 2015). In one of these trials Onyx-015 was combined with Etanercept (Enbrel) that act as an anti-inflammatory drug and inhibitor of tumour-necrosis factor (TNF) (Nemunaitis et al., 2007). Nine patients with different types of cancer including two patients with metastatic BC were enrolled and administered Onyx-015 intravenously on days 1, 8 and 15 of a 3-weeks cycle at different dose levels (1 × 10^10^, 1 × 10^11^, and 1 × 10^12^ viral particles (vp) per injection). Etanercept was administered before and during the first viral cycle. All nine patients developed grade 1/2 fever 24 h after administration of the virus with no significant adverse events demonstrating clinical safety. Both BC patients showed progressive disease (PD).

More recent findings demonstrated that E1B55K is also essential for viral mRNA export and mutants lacking this protein therefore had decreased viral fitness explaining the disappointing efficacy in the clinic with Onyx-015 (O'Shea et al., 2004; O'Shea et al., 2005). Furthermore, the deletion of the immunoregulatory genes in the E3B domain were also found to decrease viral spread in tumours in patients due to rapid viral removal by macrophages (Wang et al., 2003). These early studies clearly demonstrated the safety of OAds and that more selective gene-deletions were necessary in future developments of cancer-specific mutants to enhance viral potency.

### Deletion of the pRb-Binding E1ACR2 Domain

Because of the disappointing clinical outcomes with Onyx-015 and other E1B55K-deleted mutants the focus has since been on improving the efficacy of OAds by deleting the E1ACR2 domain (24 amino acids). Deletion of E1ACR2 prevents replication in normal healthy cells but is redundant in cancer cells with deregulated cell cycle and growth control. In contrast to the E1B55K-deleted mutants the E1ACR2-deleted variants retain high viral potency (Heise et al., 2000). To date several OAds with E1ACR2-deletions have been evaluated in clinical trials and shown to be safe and significantly more efficacious than the E1B55K-deleted mutants (Fueyo et al., 2000; Lang et al., 2018). One of these promising mutants is ICOVIR-7 that also has a modified E2F1-promotor replacing the E1A-promoter in addition to an RGD-4C motif in the HI-loop of the fibre domain to enhance tumour selectivity, integrin-binding and entry into tumour cells (Nokisalmi et al., 2010). ICOVIR-7 was evaluated in a phase I trial targeting solid tumours in 21 patients including three BC patients with the virus delivered intratumourally at escalating doses from 2 × 10^10^ to 1 × 10^12^ vp/administration. Only self-limiting grade 1/2 side effects occurred such as fever, fatigue, elevated liver transaminases and anaemia without serious adverse effects demonstrating the safety of ICOVIR-7 at high doses. Interestingly, one of the BC, one prostate cancer and one ovarian-cancer patient, showed a decrease or stabilization in tumour markers suggesting anti-tumour efficacy (Nokisalmi et al., 2010). An improved mutant based on ICOVIR-7 is VCN-1 that was evaluated in clinical trials targeting pancreatic cancer patients and reported to have direct antitumour effects including the tumour stroma (Bazan-Peregrino et al., 2021). VCN-1 expresses Hyaluronidase that contributes to the degradation of the tumour stroma to facilitate the dense TME but has not yet been evaluated in BC patients.

### Tumour-Specific Promoter-Driven OAds

Replacement of the E1A-promoter with tumour specific promoters is another approach used alone or in combination with specific gene deletions. OBP-301 (Telomelysin) is an HAdV5-based OAd with the human telomerase reverse transcriptase promoter (hTERTp) replacing the E1A-promoter (Nemunaitis et al., 2010; Yano et al., 2015). The hTERTp is highly active in cancer cells, but not in normal cells. Additional modifications in OBP-301 are replacement of the E1B genes with the internal ribosomal entry site (IRES) to improve specificity (Nemunaitis et al., 2010). OBP-301 was evaluated in a phase I clinical trial including 16 patients of which one was a BC patient (Nemunaitis et al., 2010). Administration of 1 × 10^10^, 1 × 10^11^, and 1 × 10^12^ vp once/treatment group resulted in only grade 1/2 side effects such as fever, fatigue, and chills, demonstrating safety in all patients. Viral replicative activity was observed in a subset of patients, including the BC patient, who was given a dose of 1 × 10^12^vp intratumourally. In ongoing Phase I/II trials a second intratumoural or intravenous injection is administered in combination with radiation, chemotherapy and/or immunotherapy (NCT04685499; NCT03921021; NCT04391049). However, a challenge to this strategy is to find the right balance between the antiviral response and the antitumour immunity (Sato-Dahlman et al., 2020). Examples of additional tumour-specific promoter-driven OAds are shown in Table 3 and clinical trials with reported outcomes in BC patients are listed in Table 4.

**TABLE 3:** Tumour-specific promoter-driven oncolytic adenoviruses (OAds) used in preclinical and clinical trials.

OAd	Promoter	Cancer Type	Clinical Trial status	Clinical Trial NCT number
ICOVIR-5	E2F1 promoter	Solid tumours	Completed Recruiting Not yet recruiting	NCT01844661 NCT01864759 NCT04758533 NCT05047276

VCN-01		Advanced solid tumours	Completed Recruiting Not yet recruiting	NCT02045602
				NCT02045589
				NCT03799744
				NCT05057715
				NCT03284268
CG0070		Bladder cancer	Completed Recruiting	NCT02365818
				NCT04452591
				NCT04387461
				NCT04610671
SynOV1.1	AFP promoter	Hepatocellular Carcinoma	Not yet recruiting	NCT04612504
OBP-301	hTERT-promoter	Carcinomas, melanomas and advanced solid tumours	Recruiting Active	NCT04391049
				NCT03921021
				NCT04685499
				NCT03172819
				NCT03213054
				NCT03190824
				NCT02293850
CRAd-S-pk7	Survivin-promoter	Recurrent High-Grade Gliomas	Not yet recruiting	NCT05139056
AdVince	CgA-promoter	Neuroendocrine Tumours	Recruiting	NCT02749331

**TABLE 4:** Oncolytic adenoviruses (OAds) in clinical trials including breast cancer patients.

OAds	Genetic modification	Clinical Trial results	References
Onyx-015	Deletion: E1B55K and E3B	Two patients: one PD on day 125, one discontinued	Nemunaitis et al. (2007)
ICOVIR-7	E2F-1 promoter E1ACR2 deletion RGD-4C motif in fibre	Three patients: one with stabilized tumour markers	Nokisalmi et al. (2010)
OBP-301 (Telomelysin)	hTERTp replacing E1A promoter IRES replacing E1B genes	One patient: detectable virus replication	Nemunaitis et al. (2010)

## Preclinical Studies in Models of BC

### rAd-sTRII

The rAd-sTRII OAd is an HAdV5 mutant with two deletions in the E1A gene and expression of a soluble 159-amino-acid sequence of transforming growth factor-β type II receptor (sTGFβRII) in the E3B domain (Table 5) (Wang et al., 2006). The E1A deletions include amino acid sequences 4–25 and 111–123 that are essential for binding to the p300/CREB-binding protein (CBP) and to pRb proteins, respectively. The rAd-sTRII was demonstrated to be highly cytotoxic in a dose-dependent manner in the TNBC cell line MDA-MB-231 (Wang et al., 2006). Cells infected with rAd-sTRII showed inhibition of TGF-β signalling by producing sTGFβRII without affecting the replication potential of the virus.

**TABLE 5:** Oncolytic adenoviral mutants in promising preclinical studies in TNBC models.

OAds	Genetic modification	Preclinical results	References
rAd-sTRII	Two E1A deletions prevent binding to CBP and pRb proteins sTGFβRII insertion in E3B	Inhibits TGFβ signalling and cancer cell growth	Wang et al. (2006)
rAd.DCN	hTERT promoter driving E1A GM-CSF and IRES replacing E1B19K	Inhibits tumour growth Prevents lung metastasis	Zhao et al. (2019)
SG400-E2F/IL-15	E2F-1 driving E1A IL-15 insertion in E3	Selective cancer cell killing	Yan et al. (2019)
Ad5-10miR145T	10 tandem repeats of miR-145 binding sites downstream of E1A Insertion of IRES and deletion of E3	E1A expression prevented by miR-145 Selective cancer cell killing	Shayestehpour et al. (2017)

GM-CSF, Granulocyte-macrophage colony-stimulating factor; IRES, internal ribosome entry site.

### rAd.DCN

The rAd.DCN mutant has the hTERT promoter replacing the E1A promoter, the same amino acid deletions as described in rAd-sTRII above, as well as expression of the decorin gene (Table 5) (Zhao et al., 2019). It was suggested that decorin (DCN) inhibits TGF-β signalling and inhibit metastasis, angiogenesis, and other pathophysiological processes (Isaka et al., 1996). The rAd.DCN OAd was administered intratumourally and intravenously to mice with TNBC xenografts on two occasions (Zhao et al., 2019). The authors reported significant inhibition of tumour growth for both modes of administration. Furthermore, after intravenous administration development of lung metastasis was prevented. It was reported that rAd.DCN inhibited tumour growth and metastasis as a consequence of decorin interfering with wnt/β-catenin, vascular endothelial growth factor (VEGF), the Met pathways and by modulating anti-tumour inflammatory and immune responses (Zhao et al., 2019).

### SG400-E2F/IL15

SG400-E2F/IL15 is based on HAdV5 with the E2F-1-promoter replacing the E1A-promoter and interleukin-15 (IL-15) coding sequence inserted into the E3 region for selectivity and efficacy, respectively (Table 5) (Yan et al., 2015; Yan et al., 2019). The transcription factor E2F-1 is frequently overexpressed in BC cells and predicts poor prognosis (Johnson et al., 2016). IL-15 is an immune regulator that prevents cancer cell proliferation by activating natural killer (NK) cells and CD8^+^ memory T-cells (Gillgrass et al., 2014). SG400-E2F/IL-15 was demonstrated to strongly inhibit tumour growth both in the cultured TNBC MDA-MB-231 cells and in mice with MDA-MB-231 xenografts (Yan et al., 2019).

### Ad5-10miR145T

A different approach was used when constructing Ad5-10miR145T by inserting several binding sites for a tumour suppressor miRNA (miRNA-145; miR-145) to regulate E1A expression (Table 5) (Bader et al., 2010; Ji et al., 2017; Shayestehpour et al., 2017). It has been demonstrated that miR-145 acts as a tumour suppressor and is frequently downregulated in BC (Eades et al., 2015; Zhao et al., 2016; Ding et al., 2017; Shayestehpour et al., 2017). The Ad5-10miR145T mutant carries 10 binding sites for miR-145 downstream of E1A that do not affect viral replication when miR-145T is absent. After infection with Ad5-10miR145T in TNBC and normal epithelial breast cells (HMEpCs), the E1A gene expression was reduced in HMEpCs since they express high levels of miR-145 and consequently, viral replication was prevented. In contrast, Ad5-10miR145T potently replicated and killed all tested BC cells including MDA-MB-453, MCF-7, and BT-20 that do not express miR-145 (Shayestehpour et al., 2017). Considering that MDA-MB-453 and BT-20 cells are derived from TNBC and that miR-145 has been reported as downregulated in TNBC (Eades et al., 2015), Ad5-10miR145T may be a safe and promising therapeutic for TNBC.

### Deletions of Immunomodulatory Viral Genes

Cancer cell killing by OAds is mediated by both virus-induced cell lysis and recruitment and activation of the host anti-tumour immune responses (Shaw and Suzuki, 2019). The massive lysis of cancer cells causes release of novel TAAs that in turn attract and activate host APCs, dendritic cells (DC) and cytotoxic T-cells. The viral immunomodulatory E3gp19K protein prevents MHC class I presentation of antigens and deletion of this protein promotes T-cell activation and has therefore been deleted in numerous OAds (Halldén et al., 2003; Wang et al., 2003; Man et al., 2018). For example, ORCA-010 has a single-base mutation (T29183) in E3gp19K resulting in increased membrane permeabilisation and enhanced release of progeny virions and TAAs from infected cells (Dong et al., 2014). ORCA-010 is E1ACR2-deleted with an inserted RGD-4C motif in the fibre for improved integrin binding and has demonstrated efficacy in prostate, lung and ovarian cancer models.

## Promising Combinations of OAds With Current Clinical Therapeutics

### PARP Inhibitors

The enzyme poly (ADP-ribose) polymerase (PARP) one binds to single-strand DNA breaks (SSBs) and catalyses the formation of linear and branched poly (ADP-ribose) (PAR) chains that in turn recruit additional DNA repair proteins (Zaremba and Curtin, 2007; Underhill et al., 2011). Since 2005, PARP inhibitors (PARPi) have been extensively developed to treat BRCA deficient cancers, including TNBC (D'Andrea, 2018; Farmer et al., 2005; Bryant et al., 2005). The efficacy of PARPis in BRCA-deficient BC is because unrepaired SSBs lead to double strand breaks (DSB) that normally would be repaired by homologous recombination (HR) that is not functional in BRCA deficient cancers. In BRCA1/2 deficient cells, non-homologous end joining (NHEJ) or single-strand annealing (SSA) may occur that are both error-prone, leading to complex chromatid rearrangements and eventually, to apoptosis (D'Andrea, 2018) (Underhill et al., 2011). In addition, PARPis inhibitors function as chemosensitisers by enhancing the responses to chemotherapy by preventing repair of damaged DNA (Dréan et al., 2016).

In 2018, Olaparib became the first PARPi approved by the FDA for the treatment of metastatic BC and later the same year Talazoparib was approved by both the FDA and European Medicines Agency (EMA) to treat germline BRCA mutated HER2-negative locally advanced or metastatic BC (Robson et al., 2017; Ettl et al., 2018; Litton et al., 2018; Cortesi et al., 2021). When compared with standard chemotherapy, Olaparib showed longer progression-free survival (PFS) (7 vs. 4.2 months), better response rate (59.9 vs. 28.8%), less toxicity (36.6 vs. 50.5% of grade 3 or higher adverse events), and fewer patients stopped treatment because of toxicity (4.9 vs. 7.7%; (Robson et al., 2017). Talazoparib, when compared with standard chemotherapy, showed longer PFS (8.6 vs. 5.6 months), higher objective response rate (62.6 vs. 27.2%), significant overall improvements, and significant delays for clinically meaningful deterioration (Litton et al., 2018).

### PARP Inhibitors Combined With Oncolytic Viruses

In a preclinical model of anaplastic thyroid carcinoma, the OAd *dl*922-947 (deleted in E1ACR2 and E3B) was combined with Olaparib (Passaro et al., 2015). It was demonstrated that virus-induced DNA damage was not repaired leading to increased cell death and potent *dl*922-947 replication and oncolytic activity suggesting the feasibility of PARPis and OAds as an improved therapy that warrants further investigation.

### EGFR as a Therapeutic Target

The epidermal growth factor receptor 1 (EGFR1; ErbB) is overexpressed in up to 70% of TNBC (Ueno and Zhang, 2011; Maennling et al., 2019). It has been established that inhibition of BRCA1 leads to upregulation of EGFR in mammary epithelial cells (MECs) (Burga et al., 2011). Because BRCA1 is frequently non-functional in TNBC, this likely contributes to the upregulation of EGFR. It was demonstrated that administration of erlotinib, an EGFR inhibitor, prevented growth but did not eliminate BRCA1-related breast cancers (Burga et al., 2011). Currently the EGFR is therapeutically targeted by monoclonal antibodies (mAbs) against the extracellular domain of EGFR, or by tyrosine kinase inhibitors (TKIs) that bind to the ATP pocket and therefore, prevent signal transduction (Guerrab et al., 2016; Nakai et al., 2016). However, outcomes from clinical trials using TKIs for TNBC have been surprisingly disappointing (Nakai et al., 2016; Shi et al., 2018). In a Phase II clinical trial, 115 patients with metastatic BC received cisplatin plus the EGFR inhibitor cetuximab and 58 patients received cisplatin alone (Ueno and Zhang, 2011; Baselga et al., 2013). The combination treated group showed better outcomes than with cetuximab alone, with an overall response rate of 20 vs. 10%, a PFS of 3.7 vs. 1.5 months, and an overall survival (OS) of 12.9 vs. 9.4 months.

### EGFR Targeting in Combination With Oncolytic Viruses

The OAd ICOVIR15-cBiTe, armed with an EGFR-targeting bispecific T-cell engager, demonstrated good antitumour efficacy in human lung adenocarcinoma and epidermoid carcinoma cell lines (Fajardo et al., 2017; Barlabé et al., 2020). The bispecific T-cell engager (BiTE) was composed of two single chain antibodies (scFV) that targeted the tumour-specific EGFR and CD3 on the T-cell receptor to activate T-cells and bring the complex close to the tumour cell for improved elimination. The EGFR mutant EGFRvIII is frequently expressed in glioblastomas and is specifically expressed in the cancer cells. Targeting of the EGFRvIII was exploited to engineer an OAd to target the receptor and was reported to the show selective and potent anti-glioma effects (Piao et al., 2009). Although EGFRvIII expression in BC, specifically in TNBC, is less common, an EGFRvIII-retargeted OAd may be a therapeutic approach for TNBC cases with this mutation (Del Vecchio et al., 2012).

### Androgen Receptor as a Therapeutic Target

The androgen receptor (AR) is a member of the nuclear steroid hormone receptor family. This family also includes ER and PR, and although the roles of these receptors are well established in BC, little is known about the biological functions of AR in TNBC (Rampurwala et al., 2016; Hwang et al., 2019). In a gene expression-profile analysis from 21 data sets containing 587 TNBC cases, six subtypes of TNBC were identified including the luminal AR subtype (LAR) (Lehmann et al., 2011). AR was also expressed in additional subtypes in up to 75% of all TNBCs (Hwang et al., 2019). However, the LAR subtype was found to be dependent on AR signalling with high levels of AR expression (Rampurwala et al., 2016). It was demonstrated that LAR cell lines were sensitive to the AR antagonist bicalutamide and even more sensitive to enzalutamide (Lehmann et al., 2011; Cochrane et al., 2014).

In a Phase II clinical trial using bicalutamide in patients with AR-positive and ER/PR-negative metastatic BC the most commonly reported adverse events were fatigue, hot flushes, limb oedema, and aspartate aminotransferase (AST) and alkaline aminotransferase (ALT) elevation (Gucalp et al., 2013). The PFS was 12 weeks, and a 6-months clinical benefit rate of 19% was reported for bicalutamide given at 150 mg/day. Enzalutamide was evaluated in a Phase II clinical trial in patients with AR-positive TNBC (Traina et al., 2018). The evaluable subgroup, which included patients with AR expression of ≥10% by IHC, showed an increase in OS of 4.9 months compared to the non-enzalutamide treated group (Traina et al., 2018).

A phase I trial with the selective CYP17 and AR inhibitor Seviteronel showed good tolerance to a once-daily dose of 450 mg (Bardia et al., 2018). AR targeting on its own have proven good tolerance and less toxicity than chemotherapy. However, further research is needed in order to gain a better understanding of efficacy of anti-androgens in TNBC.

### AR Targeting in Combination With OAds

Numerous OAds with AR response elements (AREs) have been developed for evaluation in prostate cancer patients (Sweeney and Halldén, 2016). The AREs typically replace the E1A-promoter to drive tumour-specific viral replication. The most frequently used ARE is from the regulatory domain of the prostate-specific antigen (PSA). One of the first developed ARE-driven OAds CG7060, also known as CN706 or CV706, showed up to 7-fold increases in replication in the presence of androgens in AR-positive cell lines (Rodriguez et al., 1997). In a Phase I clinical trial with CV706 both safety and significant reduction of PSA levels (up to 65%) was reported in prostate cancer patients (DeWeese et al., 2001).

The AR mutant (C685Y) often expressed in late-stage cancer, is activated by both androgens and anti-androgens and was utilised to engineer a novel OAd (Johnson et al., 2013). The receptor coding region was fused to the viral E1A gene to increase specific viral oncolysis. This AR-driven mutant was reported to have significantly enhanced viral activity in the presence of bicalutamide both *in vitro* and *in vivo,* suggesting a promising novel therapy for both prostate cancer and AR-positive TNBC patients.

## Immunotherapy for TNBC

Novel immune therapeutics have been developed as promising treatments for numerous cancer types including TNBC (Lee and Marks, 2010; Han et al., 2020; Mediratta et al., 2020). Currently the most promising agents are the immune checkpoint inhibitors that prevent the inhibition of cytotoxic T-lymphocyte (CTL) activity and reactivate the host anti-tumour defences (Pardoll, 2012; Navarrete-Bernal et al., 2020).

### PD-1/PD-L1 Antibodies

Programmed cell death 1 (PD-1; CD279) is a 288 amino acid transmembrane protein expressed on activated CD4^+^ and CD8^+^ T-cells, B-cells, NK-cells, monocytes, and dendritic cells (DCs) (Zajac et al., 1998; Bardhan et al., 2016; Kurachi, 2019; Seliger, 2019; Han et al., 2020). T-cell receptor activation is suppressed when PD-1 on T-cells binds to the ligands PD-L1 or PD-L2, negatively regulating the immune response (Zak et al., 2015; Vikas et al., 2018). PD-L1 is expressed on T- and B-cells, macrophages, DCs, bone marrow-derived mast cells, mesenchymal stem cells, and non-hematopoietic cells (Yamazaki et al., 2002; Riella et al., 2012; Bardhan et al., 2016). It has been established that PD-L1 is overexpressed in numerous cancers such as glioblastoma, lymphoma, melanoma, ovarian, and BC, among others that result in inhibition of the immune response in the TME (Matsuzaki et al., 2010; Muenst et al., 2013; Sun et al., 2014; Hawkes et al., 2015). High expression levels of PD-L1 correlates with poor prognosis and targeting the PD-1/PD-L1 pathway is a promising therapeutic approach (Sakuishi et al., 2010; Sabatier et al., 2015).

Pembrolizumab is an anti-PD-1 antibody that was approved by the FDA in 2014 for patients with advanced melanoma and is one of the most researched immune-checkpoint-targeted therapies (Vikas et al., 2018). In a phase II clinical trial, pembrolizumab was administered alone intravenously in patients with metastatic TNBC resulting in an objective response rate (ORR) of 5.7%, including two patients with complete response (CR) and four patients with partial response (PR). Adverse events (AE) were observed in 60.6% of patients, of which 12.9% were grade 3 or 4 (Adams et al., 2019). Although the ORR was lower than single-agent chemotherapy, treatment with pembrolizumab had less toxicity and showed durable responses.

Another FDA approved anti-PD-L1 antibody, Atezolizumab, was approved in 2016 for patients with urothelial carcinoma and is now also approved for TNBC by FDA. In a phase III clinical trial, 902 patients with TNBC whereof 451 patients received atezolizumab plus nab-paclitaxel, and 451 patients received placebo plus nab-paclitaxel (Schmid et al., 2020). The OS for PD-L1-positive patients was 25 months for the atezolizumab group vs. 18 months for the nab-paclitaxel alone group suggesting a promising outcome for the combination with standard-of-care in TNBC patients.

### Pembrolizumab in Combination With Oncolytic Viruses

Pembrolizumab has been combined with several oncolytic viruses, including OAds, in both preclinical studies and clinical trials targeting different cancer types (Chen et al., 2018). Pembrolizumab in combination with ONCOS-102 was reported to have synergistic antitumour effects in mouse models of malignant melanoma (Kuryk et al., 2018). DNX-2401 plus pembrolizumab treatment was reported to be safe in glioblastoma patients. Phase I and II ongoing clinical trials are evaluating OBP-301 OAds combined with pembrolizumab in treatments of patients with advanced solid tumours, and patients with inoperable, recurrent, and progressive squamous cell carcinoma of the head and neck (NCT03172819; NCT04685499).

### CTLA-4 Antibodies

CTLA-4 is a CD28-homolog cell surface glycoprotein expressed on regulatory T-cells (Treg) and is upregulated on activated CD4^+^ and CD8^+^ T-cells. B7-1 and B7-2 bind to CTLA-4 and CD28 (Buchbinder and Desai, 2016; Mediratta et al., 2020). Binding of B7 to CD28 molecules leads to T-cell proliferation, differentiation and cell survival while binding of B7 to CTLA-4 leads to inhibitory signals. CTLA-4 is often upregulated in cancer patients and is therefore an attractive target to enhance T-cell antitumour activity (Navarrete-Bernal et al., 2020). Ipilimumab is an approved anti-CTLA-4 antibody for the treatment of several cancers in combination with nivolumab (anti-PD-1 antibody) (Mediratta et al., 2020). Currently, there are at least three ongoing clinical trials studying the use of ipilimumab in combination with different therapeutic agents for advanced malignancies, including TNBC (NCT03126110; NCT03752398; NCT03546686).

### CTLA-4 Antibodies in Combination With Oncolytic Viruses

Treatments combining CTLA-4 antibodies and oncolytic viruses have been reported although, not yet for OAds (Engeland et al., 2014; Puzanov et al., 2016; Peters et al., 2019; Liu et al., 2021; Sugawara et al., 2021). In one interesting study, Maraba rhabdovirus was tested in TNBC-like murine models with virus administered twice intratumourally followed by five administrations of anti-CTLA-4 and anti-PD-1 antibodies intraperitoneally. A meaningful reduction in tumour growth was reported in animals treated with the combinations (Bourgeois-Daigneault et al., 2018).

## Barriers to the Systemic Delivery of OAds to TNBC Patients

Although intratumoural injection of OAds has been assessed and has been feasible in different solid tumour contexts, only intravenous systemic delivery of OAds will be able to reach distant metastatic disease. As up to 46% of TNBC patients have distant metastases, strategies to overcome the barriers to successful systemic delivery need to be overcome in order to improve efficacy across all sites of a patient’s disease (Yin et al., 2020).

One of the immediate hurdles that OAds encounter upon entering the blood stream is the presence of neutralising antibodies (NAbs) that sequester the virus rendering them unable to reach tumour sites. Around 85% of the population harbour NAbs against Ad5 OAds owing to previous natural infections meaning that this is a pertinent factor to overcome (Mast et al., 2010). Aside from NAbs, red blood cells (erythrocytes) are also able to bind OAds via both CAR and complement receptor-1. NAb and erythrocyte binding will therefore reduce the bioavailablity of circulating virus although there are certain strategies that can overcome this including adenovirus nanocomplexes (Park et al., 2010), modification of the viral coat proteins with liposome complexes (Han et al., 2008), and the use of tumour-infiltrating lymphocytes (TIL) or mesenchymal stem cells as carriers (Barlabé et al., 2020; Santos et al., 2021).

Free OAd particles within the blood that avoid NAb and erythrocyte binding are, however, highly susceptible to clearance from the systemic circulation. A major contributor to this process is the hepatic reticuloendothelial system, containing phagocytic Kupffer cells that can eliminate up to 90% of administered OAds within minutes (Parker et al., 2006; Carlisle et al., 2009; Parker et al., 2009). One strategy to help ablate the hepatic binding of OAds involves the modification of adenoviral hexon proteins, such as substitution with the adenoviral serotype 3 hexon (Short et al., 2010). A more recent strategy involves the insertion of specific amino acid peptides into the fibre knob protein of the OAd. This strategy involves two concepts: firstly, the native fibre knob is disrupted by the peptide insertion rendering it unable to bind to CAR, αvβ3/5 and coagulation factor 10 (FX), thus reducing its sequestration in the blood and non-tumour sites. Secondly, the peptides can be designed to target other tumour-specific binding sites such as αvβ6 (A20 peptide) (Uusi-Kerttula et al., 2018) and (Man et al., 2018).

Aside from vascular and hepatic sequestration, another consideration for systemic OAd therapy is the location of tumours and metastatic sites. Like many solid cancer types, the first sites of metastatic spread from primary TNBC tumour are the lungs (41%), liver (29%) and bone (24%). However, when factoring in total metastatic burden over the total disease course, 46% of patients were noted to have at least one lesion within the central nervous system (CNS) which is not the case across all solid tumour types (Lin et al., 2008). The relatively common occurrence of CNS metastases in TNBC is a significant challenge due to the fact that systemic therapies must cross the blood-brain barrier (BBB), which is particularly resistant to the transport of most small polar molecules, macromolecules and therapeutic agents (Tang et al., 2007). One strategy for metastatic CNS TNBC in mice, albeit using oncolytic herpes simplex virus (HSV), involved the intra-arterial infusion of hyperosmolar mannitol to transiently disrupt the BBB during the infusion of oncolytic HSV. The mannitol infusion allowed for extensive trafficking of the virus to intracerebral tumours compared to PBS infusion controls however this is a fairly invasive procedure (Liu et al., 2005). Alternatively, oncolytic recombinant poliovirus was delivered intrathecally to rats for the treatment of human glioblastoma multiforme, increasing survival without toxicity (Ochiai et al., 2006). An exciting new approach using an engineered oncolytic adenovirus (CRAd-S-pk7) in a phase 1 clinical trial for malignant glioma whereby neural stem cells (NSCs) were used as a vehicle to cross the BBB due to their natural tumour tropism. This trial involved intracranial delivery however systemic intravenous delivery has been efficacious in mouse models. This trial demonstrated safety and tolerability of NSC-OAd delivery even when combined with neurosurgical resections and chemoradiotherapies, and is an exciting potential candidate for treating metastatic CNS TNBC patients with systemic OAds (Barish et al., 2017; Fares et al., 2021).

## Discussion and Concluding Remarks

TNBC tumours are characterised by the absence of the ER/PR hormone receptors and HER2, and short overall survival with rapid metastasis and recurrence (Collignon et al., 2016). New agents to target this type of cancer remains an important challenge since current standard-of-care chemotherapeutics are not curative. OAds have the potential to benefit TNBC patients due to their completely different mechanisms of action that can overcome drug resistance and provide long-lasting protection through activation of the host anti-tumour immune responses (Shaw and Suzuki, 2019).

Although clinical trials using OAds such as Onyx-015, ICOVIR-7 and OBP-301 have shown good efficacy in solid cancers, improved efficacy is necessary to eliminate BCs including TNBCs (Nemunaitis et al., 2007; Nemunaitis et al., 2010; Nokisalmi et al., 2010). Novel engineered OAds have shown promise for TNBC patients in preclinical studies. The SG400-E2F/IL-15 mutant selectively and effectively infected and killed BC cells (Yan et al., 2019). The Ad5-10miR145T mutant also infected and killed BC cells, leaving normal cells unharmed (Shayestehpour et al., 2017). Ad5-10miR145T proved highly efficacious *in vivo* in TNBC tumour xenografts in mice. Because miR-145 is frequently downregulated in TNBC the Ad5-10miR145T comprise a promising new agent to be evaluated in clinical TNBC.

Recently an OAd was developed that targets pancreatic cancer through αvβ6-integrin-mediated binding and uptake, Ad5-3Δ-A20T (Man et al., 2018). The highly potent mutant harbour deletions in the E1B19K and E1ACR2, the E3gp19K for optimal replication-selectivity and immune stimulation, respectively. Ad5-3Δ-A20T is highly selective for αvβ6-integrin-expressing pancreatic cancer cells that express the integrin in high levels and effectively eliminates the cells both as a single agent and in combination with the chemotherapy drug gemcitabine. Ad5-3Δ-A20T also has a higher efficacy after systemic delivery than the corresponding parental virus in mice with PDAC xenografts indicating a longer half-life in blood (Stella Man et al., 2019). Since αvβ6-integrin is expressed only in solid tumours, including TNBC cell lines and tumours, and not in healthy tissues (Whilding et al., 2017), we suggest that Ad5-3Δ-A20T has potential for translation into the clinic for treatment of TNBC. We recently demonstrated that the replication and anti-tumour efficacy of OAds including Ad5-3Δ-A20T can be augmented when combined with novel therapeutics such as histone deacetylase inhibitors (HDACis) (Rodríguez et al., 2022).

Small molecule anti-cancer drugs that target the dysregulated pathways in TNBC have been shown to improve efficacy in combination with oncolytic viruses. For example, inhibitors of EGFR and AR in combination with OAds are anticipated to have good responses in TNBC through synergistic effects on multiple cellular pathways (Piao et al., 2009; Johnson et al., 2013). Combining the small molecule PARP inhibitor Olaparib with the OAd *dl*922-947 resulted in significantly improved efficacy in anaplastic thyroid carcinoma models (Passaro et al., 2015). The main reason for the enhanced tumour elimination is likely due to synthetic lethality, with virus causing cellular DNA damage that cannot be repaired in the presence of a PARPi mimicking the efficacy with PARPi in BRCA1/2 mutated TNBC cells. Because of the frequent BRCA mutations in TNBC, this approach is anticipated to improve clinical outcomes in clinical TNBC. Other combinations including the novel immune checkpoint inhibitors targeting PD-1/PD-L1 and CTLA-4 with OAds have shown good efficacy in several solid tumours (Engeland et al., 2014; Puzanov et al., 2016; Kuryk et al., 2018; Peters et al., 2019; Liu et al., 2021; Sugawara et al., 2021). This approach is expected to be highly efficacious due to the simultaneous release of novel cancer antigens by virus-mediated lysis and blocking of the immune checkpoints to reactivate the host anti-tumour defences.

Although great advancements are being made in the design of OAds with far more sophisticated mutants being generated, the current barriers of intravenous systemic delivery to all metastatic tumour sites cannot be ignored. Achieving sufficient bioavailability within the blood with sufficient trafficking and tumour penetrance will invariably be a bottleneck to efficacy for most OAds tested clinically. Coordinated efforts to investigate better strategies of delivering OAds to patients is equally important to their design, particularly in the context of hard-to-reach tumour sites such as those within the CNS.

To conclude, the safety of OAds have been demonstrated in thousands of patients administered various mutants using all modes of administration. Antitumour efficacy has been reported when virus was delivered intratumourally and in combination with cytotoxic drugs, small molecule inhibitors and immune checkpoint blockade. The use of OAds to treat TNBC is still in its infancy and further preclinical and clinical trials need to be performed however, promising results are to be expected.
